# Qingxuan Jiangya Decoction Reverses Vascular Remodeling by Inducing Vascular Smooth Muscle Cell Apoptosis in Spontaneously Hypertensive Rats

**DOI:** 10.3390/molecules21070956

**Published:** 2016-07-22

**Authors:** Fei Xiao, Fei He, Hongwei Chen, Shan Lin, Aling Shen, Youqin Chen, Jianfeng Chu, Jun Peng

**Affiliations:** 1 College of Pharmacy, Fujian University of Traditional Chinese Medicine, 1 Qiuyang Road, Minhou Shangjie, Fuzhou 350122, Fujian, China; ybxwdj@hotmail.com; 2 Academy of Integrative Medicine, Fujian University of Traditional Chinese Medicine, 1 Qiuyang Road, Minhou Shangjie, Fuzhou 350122, Fujian, China; hefei1288@163.com (F.H.); 15806018983@163.com (H.C.); saling86@hotmail.com (A.S.); 3 Fuqing Health and Family Planning Bureau, 23 Futang Road, Fuqing 350300, Fujian, China; 4 Fujian Key Laboratory of Integrative Medicine on Geriatrics, Fujian University of Traditional Chinese Medicine, 1 Qiuyang Road, Minhou Shangjie, Fuzhou 350122, Fujian, China; lisa3350@163.com; 5 Case Western Reserve University School of Medicine, Rainbow Babies and Children’s Hospital, Cleveland, OH 44106, USA; yxc571@case.edu

**Keywords:** Qingxuan Jiangya Decoction, vascular remodeling, vascular smooth muscle cell, apoptosis, spontaneously hypertensive rats

## Abstract

Qingxuan Jiangya Decoction (QXJYD), a traditional Chinese medicine formula prescribed by academician Ke-ji Chen, has been used in China to clinically treat hypertension for decades of years. However, the molecular mechanisms of its action remain largely unknown. In this study, we examined the therapeutic efficacy of QXJYD against elevated systolic blood pressure in the spontaneously hypertensive rat (SHR) model, and investigated the underlying molecular mechanisms. We found that oral administration of QXJYD significantly reduced the elevation of systolic blood pressure in SHR but had no effect on body weight change. Additionally, QXJYD treatment significantly decreased the media thickness and ratio of media thickness/lumen diameter in the carotid arteries of SHR. Moreover, QXJYD remarkably promoted apoptosis of vascular smooth muscle cells and reduced the expression of anti-apoptotic B-cell leukemia/lymphoma 2. Furthermore, QXJYD significantly decreased the plasma Angiotensin II level in SHR. Collectively, our findings suggest that reversing vascular remodeling via inducing VSMC apoptosis could be one of the mechanisms whereby QXJYD treats hypertension.

## 1. Introduction

Hypertension is a key risk factor for various cardiovascular diseases, such as stroke, myocardial infarction, and heart failure [1], accounting for approximately 9.4 million deaths globally each year [2]. It has been predicted that the global prevalence of hypertension will increase by 10% each year between 2000 and 2025 [3]. Hypertension is a complex and progressive condition which can arise from various genetic and pathogenic causes. Vascular remodeling is an early key outcome of hypertension, characterized by the hypertrophy of vascular smooth muscle cells (VSMCs) and increased deposition of extracellular matrix [4]. Although vascular remodeling is initially an adaptive process in response to long-term pressure overload, it contributes to the pathophysiology of vascular diseases, circulatory disorders, and organ damage [4,5]. Clinical and experimental models of hypertension have demonstrated the importance of vascular structure in the regulation of blood pressure [6,7,8]. Increased vascular mass and arterial wall rigidity accompanied by VSMC hypertrophy or hyperplasia are key contributors to hypertension [9,10,11]. Vascular remodeling is often characterized by an imbalance between proliferation and apoptosis in VSMCs [12]. Apoptosis plays an essential role during tissue morphogenesis and homeostasis by eliminating damaged or impaired cells, which is tightly regulated by many factors including anti-apoptotic B-cell leukemia/lymphoma 2 (Bcl-2) family members [13,14]. It has been shown that elevated expression of anti-apoptotic Bcl-2 and/or decreased expression of pro-apoptotic Bcl2 associated X protein (Bax) are commonly found in the arteries of spontaneously hypertensive rats (SHR; an effective model for research of hypertension), suggesting that a reduction in apoptosis of damaged VSMCs is involved in the pathogenesis of vascular remodeling [15,16,17]. Therefore, protection against vascular remodeling via promotion of VSMC apoptosis could be a potential therapeutic target for the treatment of hypertension-associated diseases [10,11].

Traditional Chinese medicines (TCM) have long been used as alternative remedies for a variety of diseases including cardio-cerebrovascular diseases [18,19,20]. TCM formula Qingxuan Jiangya Decoction (QXJYD), prescribed by academician Ke-ji Chen, has been used in China to clinically treat hypertension for decades of years. In addition, many active components identified in QXJYD, such as rhynchophylline, gastrodin, and baicalin, have been shown to possess anti-hypertension activity [21,22,23,24]. However, the molecular mechanisms of QXJYD’s action remain largely unknown. Using a SHR model, in the present study we found that QXJYD could significantly reduce elevated systolic blood pressure, demonstrating its therapeutic efficacy against hypertension. To investigate the mechanism mediating the bioactivity of QXJYD, we evaluated its effect on vascular remodeling. Our data showed that QXJYD treatment could significantly decrease the media thickness (MT) and ratio of media thickness/lumen diameter (LD) in the carotid arteries of SHR, indicating that QXJYD can reverse vascular remodeling during hypertension. Moreover, QXJYD remarkably promoted VSMC apoptosis through inhibiting Bcl-2 expression. Therefore, the anti-hypertension activity of QXJYD is probably mediated by reversing vascular remodeling via inducing VSMC apoptosis.

## 2. Results

### 2.1. HPLC Fingerprint Analysis of QXJYD

**Figure 1:**
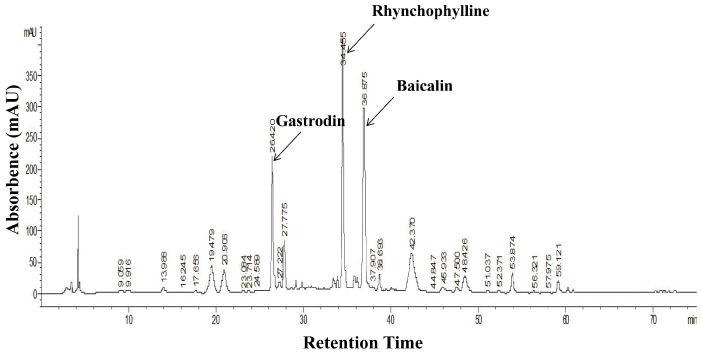
Representative Chromatographic fingerprint of QXJYD.

We prepared and analyzed the chromatographic fingerprint of QXJYD (Figure 1). We estimated gastrodin, rhynchophylline, and baicalin in QXJYD by comparison with each standard compound, which had retention times of 26.42 min, 34.45 min, and 36.87 min (see Supplementary Figure S1).

### 2.2. QXJYD Decreased Elevation of Blood Pressure in SHRs

**Figure 2:**
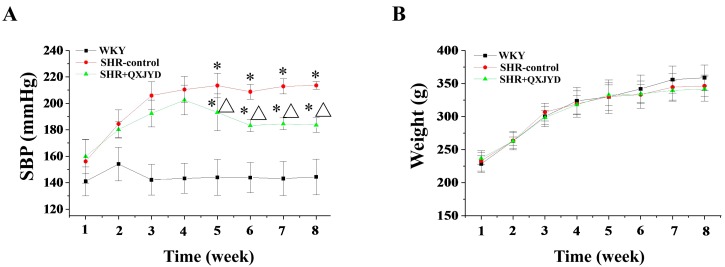
Effect of QXJYD treatment on blood pressure. (**A**) Systolic blood pressure (SBP); and (**B**) body weight were measured in spontaneously hypertensive rats (SHR-control, SHR + QXJYD) and Wistar Kyoto (WKY) rats (*n* = 10). All values were represented as mean ± SD. * *p* < 0.05, compared to WKY group; ^Δ^ *p* < 0.05, compared to SHR-control group.

The effect of QXJYD on hypertension was evaluated by measurement of systolic blood pressure (SBP) of SHRs, while its adverse effect was examined through determining body weight changes. As shown in Figure 2A, SHR displayed obvious manifestation of hypertension, as compared with Wistar Kyoto (WKY) rats. However, the elevation of SBP in SHRs was significantly reduced after treatment with QXJYD for five weeks (Figure 2A). At the end of experiment, the average SBP in SHR-control or SHR+QXJYD group was 216 ± 7 or 193 ± 8 mmHg (*n* = 10), respectively. However, administration of QXJYD did not affect body weight change in experimental rats, suggesting no toxicity (Figure 2B).

### 2.3. QXJYD Reversed Vascular Remodeling in SHRs

**Figure 3:**
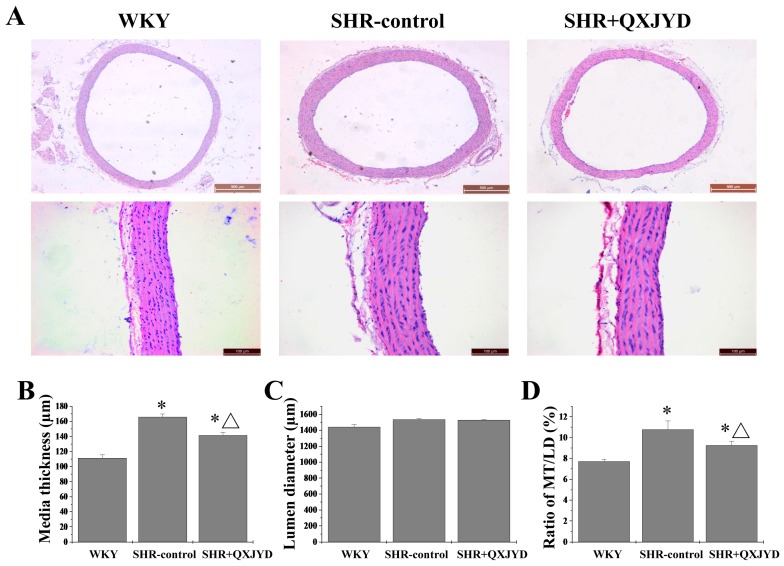
Effect of QXJYD treatment on aortic remodeling. (**A**) Histopathological changes of thoracic aorta in each group (*n* = 10) was observed by Harris Hematoxylin and Eosin (H&E) staining. Images were representatives taken at a magnification of 20× (top, scale bar = 500 μm) or 40× (bottom, scale bar = 100 μm); (**B**) Media thickness (MT); (**C**) Lumen diameter (LD); and (**D**) MT/LD was measured. All values were represented as mean ± SD; * *p* < 0.05 compared to WKY group; ^Δ^ *p* < 0.05 compared to SHR-control group.

The histological changes of thoracic aorta were determined by Harris Hematoxylin and Eosin (H&E) staining. As shown in Figure 3, although there was no significant difference in LD between the three groups, the MT of thoracic aorta in SHR-control group was significantly greater than that of WKY group, suggesting the vascular remodeling in the rats of the SHR-control group. However, the thickening of thoracic aorta in SHRs was significantly ameliorated by QXJYD treatment. The MT in WKY, SHR-control, or SHR + QXJYD group was 111 ± 4, 165 ± 4, or 141 ± 2 μm, respectively.

### 2.4. QXJYD Promoted VSMC Apoptosis in SHRs

**Figure 4:**
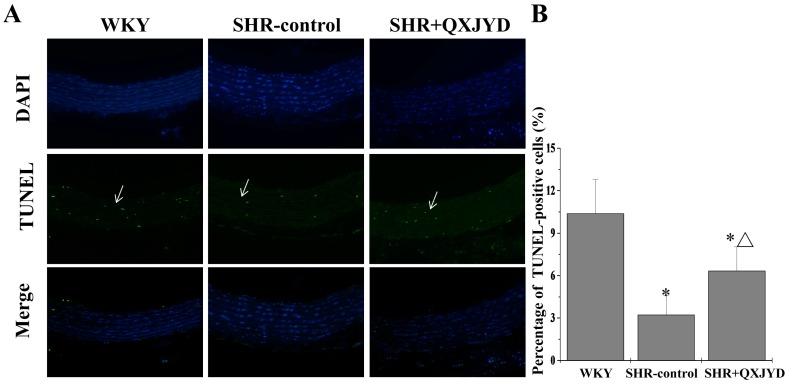
Effect of QXJYD treatment on apoptosis of VSMCs. (**A**) Thoracic aorta in each group (*n* = 10) was processed for Terminal deoxynucleotidyl transferase dUTP nick end labeling (TUNEL) analysis. Nuclei of all cells were observed through 4,6-diamino-2-phenylindole hydrochloride (DAPI) staining, while apoptotic cells were visualized by green fluorescence. Images were representatives taken by confocal fluorescence microscope at a magnification of 20×; (**B**) Apoptotic rate was shown as the percentage of TUNEL-positive cells. All values were represented as mean ± SD; * *p* < 0.05 compared to WKY group; ^Δ^ *p* < 0.05 compared to SHR-control group.

Apoptosis of aortic VSMCs was determined by terminal deoxynucleotidyl transferase dUTP nick end labeling (TUNEL) assay. As shown in Figure 4, impaired apoptosis might be associated with thickening or vascular remodeling of the thoracic aorta in SHRs, which however was reversed by QXJYD treatment. The percentage of TUNEL-positive cells in WKY, SHR-control, or SHR + QXJYD group was 10.3% ± 2.3%, 3.2% ± 1.3%, or 6.3% ± 1.7%, respectively.

### 2.5. QXJYD Inhibited Anti-Apoptotic Bcl-2/Bax Ratio in Thoracic Aorta of SHRs

**Figure 5:**
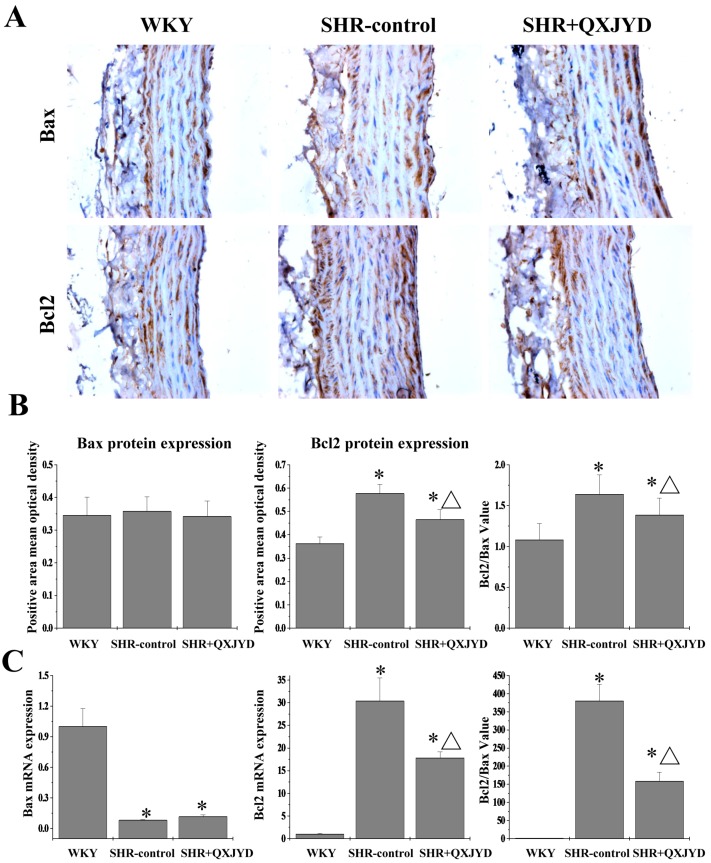
Effect of QXJYD treatment on the expression of Bax and Bcl-2 in VSMCs. (**A**) Immunohistochemistry analysis was performed to determine the protein expression of Bax and Bcl-2 in thoracic aorta from each group (*n* = 8). Images were representatives taken at a magnification of 20×; (**B**) Quantification of the mean expressions of Bax and Bcl-2 protein; (**C**) The mRNA expressions of Bax and Bcl-2 was examined by real-time PCR. All values were represented as mean ± SD; * *p* < 0.01 compared to WKY group, ^Δ^ *p* < 0.05 compared to SHR-control group.

The protein and mRNA expression levels of Bax and Bcl-2 in VSMCs were assessed by immunohistochemistry and real-time PCR analyses. As shown in Figure 5A, Bcl-2 protein expression in the SHR-control group was significantly increased, as compared to both WKY group. However, hypertension-induced Bcl-2 protein expression was profoundly suppressed by QXJYD treatment. The expression of Bax protein had no difference in all three groups. Data from real-time PCR assay showed that QXJYD significantly reduced Bcl-2 mRNA expression in SHRs, whereas the level of Bax mRNA was significantly increased after QXJYD treatment. Thus, QXJYD-induced apoptosis of VSMCs in hypertentive rats was probably regulated by the inhibition of anti-apoptotic Bcl-2/Bax ratio.

### 2.6. QXJYD Reduced Plasma Ang II Production in SHRs

**Figure 6:**
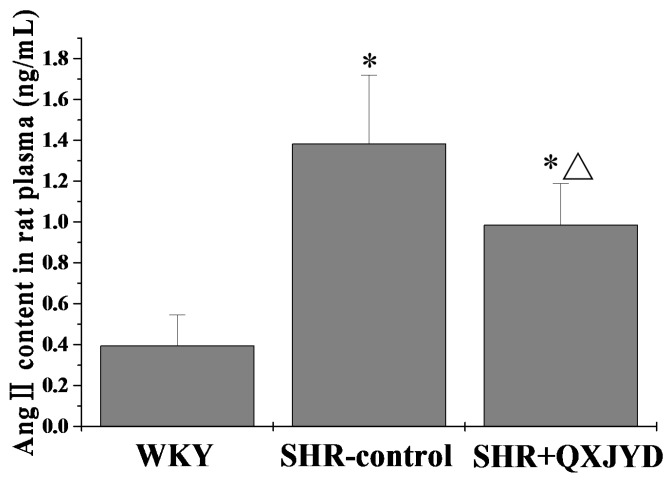
Effect of QXJYD treatment on plasma Ang II production. The level of Angiotensin II (Ang II) in plasma from each group (*n* = 10) was examined by ELISA. All values were represented as mean ± SD; * *p* < 0.01 compared to WKY group, ^Δ^ *p* < 0.05 compared to SHR-control group.

Plasma Angiotensin II (Ang II) level was determined by ELISA. As shown in Figure 6, QXJYD treatment could significantly inhibit hypertention-induced Ang II production in vivo. The plasma Ang II level in WKY, SHR-control or SHR + QXJYD group was 0.39 ± 0.15, 1.38 ± 0.33, or 0.98 ± 0.20 ng/mL, respectively.

## 3. Discussion

Natural products, including TCM, have long been used in China as important alternative remedies for various diseases. As a well-known TCM formula prescribed by Academician Ke-ji Chen, QXJYD has been used in China to clinically treat hypertension for more than 60 years. However, the precise mechanisms of how QXJYD exerts its function remain largely unclear. Therefore, before QXJYD can be further developed as an anti-hypertension agent, the underlying molecular mechanism mediating its bioactivities should first be elucidated.

Using SHR as a hypertension model here, we first demonstrated the therapeutic efficacy of QXJYD against hypertension, as evidenced by its suppressive effect on the increased SBP in SHR model. In addition, QXJYD did not show apparent toxicity since it had no effect on body weight change of experimental animals during the whole course of study. To investigate the mode of action of QXJYD we evaluated its effect on vascular remodeling which is a pathological process associated with progression of hypertension [25,26,27]. Our data clearly showed that QXJYD treatment could significantly decrease thickening of thoracic aortas in SHR model, indicating its effect of reversing vascular remodeling. The process of vascular remodeling is typically characterized by the hypertrophy of VSMCs, which partially results from the impaired apoptosis. By using TUNEL assay in the present study, we proposed that QXJYD reversed vascular remodeling probably via promotion of apoptosis in aortic VSMCs.

Apoptosis can be triggered by either intrinsic or extrinsic stimuli. The best understood intrinsic apoptotic pathway in vertebrate cells is centered at the mitochondria, which is therefore called mitochondrion-dependent apoptosis. This pathway is tightly regulated by Bcl-2 family members, functioning either as promoter (such as Bax) or as inhibitor (such as Bcl-2). Aberrant expression of Bcl-2 family proteins impairs the normal apoptotic program, resulting in many apoptosis-related diseases. Higher Bcl-2-to-Bax ratios due to the upregulation of Bcl-2 and/or downregulation of Bax expression are commonly found in VSMCs of hypertension. Here we demonstrated that QXJYD treatment suppressed the mRNA expression of Bcl-2 but increased that of Bax. In a consistent manner, QXJYD reduced Bcl-2 protein level although there was no effect on Bax protein expression. Therefore, QXJYD induced apoptosis by decreasing anti-apoptotic Bcl-2/Bax ratio in VSMCs of SHR model.

Ang II is a well-known regulator of VSMC hypertrophy. Ang II has been reported to act as a bifunctional modulator of VSMC apoptosis through either the anti-apoptotic, angiotensin type 1 receptor (AT1R), or the pro-apoptotic angiotensin type 2 receptor (AT2R) [28]. Previous studies have shown that Ang II can induce apoptosis in AT2R transfected VSMCs and protect native VSMCs against apoptosis via AT1Rs [29]. During vascular remodeling, VSMC often shows excessive proliferative and hypertrophic activities [30] through the increase in phosphorylation of extracellular signal regulated kinase 1/2 [31]. In order to evaluate Angiotensin-converting enzyme (ACE)—specifically cleaving Ang I into Ang II—inhibitory activity of QXJYD, we measured Ang II content in rats’ plasma. Results from this study indicated that QXJYD treatment significantly reduced plasma Ang II production in SHR model, which was consistent with the inhibitory effect of QXJYD on vascular remodeling and VSMC apoptosis. Components of QXJYD may inhibit ACE in vivo. Collectively, our findings suggest that reversing vascular remodeling via inducing VSMC apoptosis could be one of the mechanisms whereby QXJYD treats hypertension.

Besides Ang II, there are many other mechanisms involved in the balance of apoptosis and proliferation of VSMCs, such as NF-E2-related factor 2 (Nrf2)/Kelch-like ECH-associated protein 1 (Keap1) system [32], Phosphatase and Tensin homolog deleted on chromosome 10 (PTEN)/Protein Kinase B (AKT) pathway [33] and Ras homolog gene family, member A (RhoA)/Rho-associated protein kinase (ROCK) pathways [34]. In addition, like other TCM formulas, QXJYD is a combination of several natural products, all of which contain numerous chemical compounds including rhynchophylline, gastrodin, and baicalin [21,22,23,24]. It is unknown how many of these compounds contain anti-hypertensive activity. The signaling pathway(s) with which these components exert their function remains unknown also. These intriguing questions should be clarified by future studies before we fully explore the molecular mechanisms of therapeutic effects of QXJYD and develop better drugs for treatment of hypertension.

## 4. Materials and Methods

### 4.1. Materials and Reagents

TUNEL FITC apoptosis detection kit was purchased from Vazyme Biotech Co., Ltd. (Nanjing, China). Histostain-Plus Kit was purchased from Mai Xin biotechnology (Fuzhou, China). Bax, Bcl-2, and β-actin primers were synthesized by Invitrogen (Grand Island, NY, USA). TRIzol reagent, PrimeScript™ RT reagent Kit and SYBR Premix Ex Taq II Kit were provided by Takara Biotechnology Co., Ltd. (Dalian, Liaoning, China). Bcl-2, Bax antibodies and horseradish peroxidase (HRP)-conjugated secondary antibodies were obtained from Cell Signaling Technology (Beverly, MA, USA). Ang II rat ELISA kit was purchased from Westang Biotech Co., Ltd. (Shanghai, China). All other chemicals were obtained from Sigma Chemicals (St. Louis, MO, USA) unless otherwise stated.

### 4.2. Preparation and HPLC Analysis of QXJYD

210 g of QXJYD obtained from the Third Affiliated Hospital of Fujian University of Traditional Chinese Medicine was extracted with 1000 mL of distilled water using reflux method and was filtered twice. The water solvent was evaporated on a rotary evaporator (Yarong, Model RE-2000, Shanghai, China). The resulting solution was concentrated to produce 28.91 g dried water extract of QXJYD. 28 g of dried water extract was then re-suspended in 70 mL of 0.9% NaCl solution and stored at 4 °C for subsequent animal experiments. The remaining dried QXJYD was dissolved in 1 mL absolute methanol. In order to improve the solubility of dried QXJYD, sonication was performed for 30 min. This solution was subsequently partitioned using a separating funnel and stored at 4 °C for principal component analysis using High Performance Liquid Chromatography (HPLC).

HPLC analysis was carried using an Aglient 1200 HPLC system (Cold Spring, NY, USA). QXJYD was diluted to a final concentration of 50 mg/mL in methanol. Rhynchophylline, gastrodin, and baicalin standards were used to identify the components in QXJYD. The analysis was performed on phenomenex luna C18 reverse phase column (250 mm × 4.6 mm I.D., 5 µm). The flow rate was 1 mL/min and injection volume was 10 µL. The detection wavelength was 280.8 nm and oven temperature was set to 37 °C. The mobile phase consisted of 0.1% phosphoric acid aqueous solution (eluent A) and methanol (eluent B) with the following gradient program: 5% eluent B from 0 to 10 min; 10%–40% eluent B from 10 to 30 min, and 40%–100% eluent B from 30 to 75 min.

### 4.3. Animals

Twenty male SHRs (age: 7 weeks; weight: 200 ± 20 g) and 10 male WKYs (age: 7 weeks; weight: 200 ± 20 g) were obtained from Slac Laboratory Animal Technology Co., Ltd. (Shanghai, China). All rats were housed in clean specific pathogen-free rooms with controlled temperature (22 °C), humidity, and a 12-h light/dark cycle. Food and water were provided ad libitum throughout the experiment. All animal experiments were performed strictly in accordance with international ethical guidelines and the National Institutes of Health Guide concerning the care and use of laboratory animals, and were approved by the Institutional Animal Care and Use Committee of Fujian University of Traditional Chinese Medicine.

### 4.4. Drug Administration and Blood Pressure Measurement

After acclimation for one week, rats were divided into three groups (*n* = 10), termed as WKY, SHR-control and SHR + QXJYD. Rats in SHR + QXJYD group were orally treated with 60 mg/kg of QXJYD every day, while rats in WKY and SHR-control group were treated with saline solution. Systolic blood pressures of all rats were measured once per week, with the tail-cuff plethysmograph method using CODA™ non-invasive blood pressure system (Kent Scientific, Torrington, CT, USA).

### 4.5. Vessel Morphometry

Artery morphological characteristics were assessed using H&E Staining. Paraffin-embedded 5 µm-thick sections of aorta samples were stained with Harris Hematoxylin for 2 min then washed under running tap water 5 min. Slides were then differentiated in 1% acid alcohol 30 s, then washed under running tap water 5 min, before staining with Eosin for 1 min. Following gradient dehydration and drying, samples were measured using true color multi-functional cell image analysis system (Image-Pro Plus, Media Cybernetics, Rockville, MD, USA). The aortic media was defined as the region between the internal and external elastic laminae; the intima area was defined as the region between the endothelium and the internal elastic laminae; the lumen area was defined as the area encompassed by the endothelium. The morphometric measurements for each vessel were calculated based on the average of its proximal and distal regions. VSMCs were stained blue in the tunica media.

### 4.6. In Situ Apoptosis Detection by TUNEL Staining

5 µm-thick sections of aorta samples were analyzed using TUNEL fluorescein isothiocyanate (FITC) apoptosis detection kit (Vazyme Biotech Co., Ltd.). TUNEL-positive cells exhibited apoptosis characteristics including condensed chromatin and cellular shrinkage. Apoptotic cells were counted as FITC-positive cells (stained green) and total number of cells was counted as 4,6-diamino-2-phenylindole hydrochloride (DAPI) (stained blue) in five randomly selected fields for each slide. The percentage of apoptotic cells was calculated as the ratio of the number of TUNEL-positive cells to the total number of cells, as the mean of the five randomly selected fields. The sections of aorta samples after deparaffinage were washed with PBS and incubated for 10 min with DAPI protected from light. The cover slips were sealed onto slides with nail polish and stored at −20 °C until image collection by florescence microscopy.

### 4.7. Immunohistochemistry Analysis

Bax and Bcl-2 expression were assessed by immunohistochemistry. Briefly, 5 μm cross sections of arteries were fixed in 4% paraformaldehyde (pH = 7.4). The slides were subjected to antigen retrieval and the endogenous peroxidase activity was blocked with 3% hydrogen peroxide. Slides were incubated with rabbit polyclonal antibodies against Bax (1:200) and Bcl-2 (1:200) (Cell Signaling Technology). After washing with PBS, slides were incubated with biotinylated secondary antibody followed by conjugated HRP-labelled strepta-vidin (Maixing), then washed with PBS. The slides were then incubated with diaminobenzidine (Maixing) as the chromogen, followed by counterstaining with diluted Harris hematoxylin (Maixing). Five microscopic fields (magnification 400×) were randomly selected in each slide, and the average optical density of positive cells in each field were measured using true color multi-functional cell image analysis system (Image-Pro Plus, Media Cybernetics). To rule out any non-specific staining, PBS was used instead of primary antibody as the negative control.

### 4.8. RNA Extraction and Quantitative PCR Analysis

Total RNA was extracted using TRIzol reagent (Takara Biotechnology Co., Ltd.) according to the manufacturer’s instructions. To synthesize cDNA, 1 µg total RNA was reverse-transcribed using a Prime Script^®^ II 1st strand cDNA Synthesis kit (Takara Biotechnology Co., Ltd.). Real-time fluorescence quantitative PCR was performed using SYBR-Green premix (Applied Biosystems, Carlsbad, CA, USA) according to the manufacturer’s instructions, using the following parameters: 40 cycles; 50 °C for 2 min, 95 °C for 7 min, 60 °C for 15 s, and 60 °C for 30 s. β-actin was used as the internal control. PCR primer sequences (5′→3′) as shown in the Table 1:

### 4.9. Measurement of Ang II in Rat Plasma

The blood samples collected during exsanguination were transferred to a tube containing 10% EDTA-2K anticoagulant. The uncoagulated blood was centrifuged at 2000× *g* for 10 min at 4 °C to separate the plasma. Measurement of the total Ang II in the plasma was performed using an ELISA kit according to the manufacturer’s instructions.

### 4.10. Statistical Analysis

All data were expressed as mean ± standard deviation (SD). Statistical significance was calculated with one-way analysis of variance using SPSS software (version 18.0; SPSS, Inc., Chicago, IL, USA). *p* < 0.05 was considered as statistically significant.

## 5. Conclusions

In the present study, we demonstrated that the TCM formula QXJYD could significantly reduce the elevation of systolic blood pressure in an SHR model but had no effect on body weight changes, indicating its therapeutic efficacy against hypertension without apparent toxicity. QXJYD treatment significantly decreased the thickening of thoracic aorta in SHR model, suggesting its activity of reversing vascular remodeling during hypertension. QXJYD remarkably promoted apoptosis of VSMCs and reduced the expression of anti-apoptotic Bcl-2. Taken together, it is suggested that reversing vascular remodeling via promoting VSMC apoptosis might be one of the mechanisms by which QXJYD exerts its anti-hypertension effects.

## Figures and Tables

**Table 1:** The primer sequences for quantitative PCR analysis.

Gene Name	Sequence
β-actin	F: 5′-TGTCACCAACTGGGACGATA-3′
	R: 5′-GGGGTGTTGAAGGTCTCAAA-3′
Bax	F: 5′-TGCTACAGGGTTTCATCCAG-3′
	R: 5′-TGTTGTTGTCCAGTTCATCG-3′
Bcl-2	F: 5′-GGTGGACAACATCGCTCTG-3′
	R: 5′-ACAGCCAGGAGAAATCAAACA-3′

## Supplementary Materials

Supplementary materials can be accessed at: http://www.mdpi.com/1420-3049/21/7/956/s1.
