# Supplementary material for: Qingxuan Jiangya Decoction Reverses Vascular Remodeling by Inducing Vascular Smooth Muscle Cell Apoptosis in Spontaneously Hypertensive Rats
Source: Molecules. 2016 Jul 22;21(7):956. doi: 10.3390/molecules21070956 (PMC6274417; doi:10.3390/molecules21070956)
Supplement: Supplementary file 1 [file molecules-21-00956-s001.pdf]

Supplementary Materials: Qingxuan Jiangya Decoction Reverses Vascular Remodeling by Inducing Vascular Smooth Muscle Cell Apoptosis in Spontaneously Hypertensive Rats

Fei Xiao, Fei He, Hongwei Chen, Shan Lin, Aling Shen, Youqin Chen, Jianfeng Chu and Jun Peng

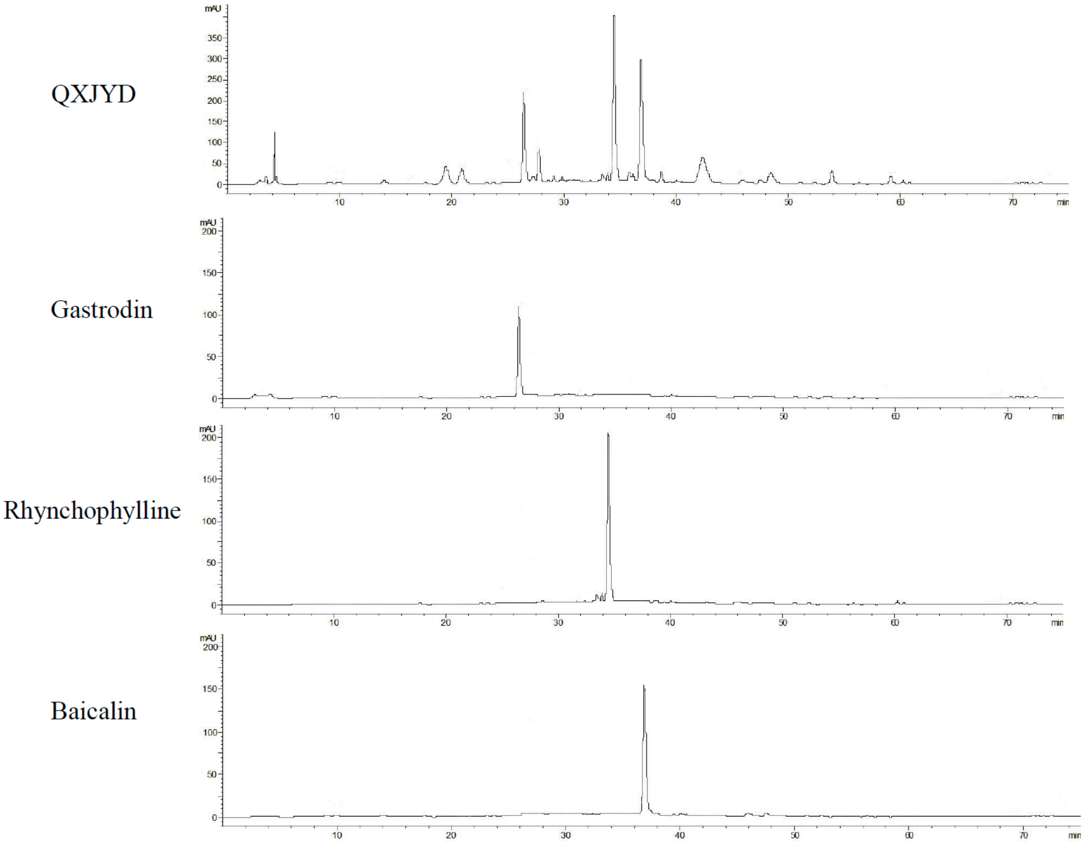

Figure S1. Chromatographic fingerprint of QXJYD and the standard compounds.
